# Engaging Aging Individuals in the Design of Technologies and Services to Support Health and Well-Being: Constructivist Grounded Theory Study

**DOI:** 10.2196/12393

**Published:** 2019-03-20

**Authors:** Vikki Du Preez, Retha De La Harpe

**Affiliations:** 1 Department of Design Faculty of Informatics and Design Cape Peninsula University of Technology Cape Town South Africa; 2 Faculty of Informatics and Design Cape Peninsula University of Technology Cape Town South Africa

**Keywords:** technology, healthy aging, grounded theory, qualitative research

## Abstract

**Background:**

Changes noted within the aging population are physical, cognitive, as well as emotional. Social isolation and loneliness are also serious problems that the aging population may encounter. As technology and apps become more accessible, many basic services, such as those offered by social services, well-being organizations, and health care institutions, have invested in the development of supportive devices, services, and Web-based interactions. Despite the perceived benefits that these devices and services offer, many aging individuals choose not to engage, or engage in a limited manner. To explore this phenomenon, we developed a theory to describe the condition for engagement.

**Objective:**

The main objective of this study was to understand the perceptions of an aging South African population regarding Web-based services and technologies that could support aging in place (AiP). Although the concept of AiP speaks to a great number of everyday activities, this paper explores aspects of health and well-being as being central to AiP.

**Methods:**

The study used a grounded theory (GT) methodology, relying on an iterative and simultaneous process of data collection, coding, category development, and data comparisons. Data were collected through qualitative methods, including interviews (13 participants aged between 64 and 85 years), 2 participatory workshops (15 participants), and observations. The study focused on Charmaz’s approach to constructivist GT, which puts forward the premise that theory or knowledge cannot take shape in a purely objective manner. Instead, theory is constructed through the interaction of the researcher and research participant. Coding and data analysis were supported with ATLAS.ti (ATLAS.ti Scientific Software Development GmbH).

**Results:**

The study resulted in a substantive theory exploring the process of interaction and engaging factors through user insights and experiences. The emerging design theory, *Ageing User Decision-Driven Engagement* (AUDDE), explored the elements that support engagement with technology and supportive apps, which could offer access to required health and wellness services.

**Conclusions:**

In AUDDE, the perceived value of the interaction is a crucial catalyst for engagement. Aging users continuously make meaning of their experiences, which affects their current and future actions.

## Introduction

### Background

Globally, health care systems have to deal with the exponential growth of the aging population, adding strain to health care service provision while still having to commit to achieving the sustainable development goal of the right to good health for all ages [1]. There is a general shift of the population toward older age being referred to as population aging [2], who not only live longer but also deal with complex interrelated factors related to their quality of life (QoL) [1-3]. At the same time, fast technology advances add pressure to the development of relevant technology-enabled health care services and the potential mediating role that technology could play toward the QoL of the older person [1]. The exponential growth of both the aging population with the complexity of health states and fast technology advances results in health care services struggling to deliver quality health services to maintain the human dignity of the aging population [4-6]. The needs and preferences of older people to enhance their intrinsic capacity to negotiate their own changing world toward new ways of functioning as part of healthy aging are complex [2,7]. Design thinking offers an approach that deals with complexity with a focus on empathy, context, ideation, and iteration as part of designing human-centered services [3].

The danger of fast technology advances is that they could potentially widen the gap between younger and older population groups, especially considering the diversity and multitude of cognitive and physical abilities and health status associated with aging groups, which may make adoption of technology more difficult [3]. Efforts should be made to include the older users in the service design and implementation processes, owing to their emergence as important consumers of services rather than merely regarding them as passive recipients of technology [1,8]. The design of fit for purpose service should focus on integrated care that is person-centered and empowers the care recipient for the older user to continue to contribute to society [7]. As a recipient of care services, it is important to understand the older users’ experience in their own situated context [9]. *Gerontechnology* is a concept for considering the impact of technology on the QoL of the aging population, where technology has the ability to enable services [6]. When considering the relationship between technology and aging, it is important to regard older persons as active consumers of technology-enabled services and active cocreators of technology during the design process [8].

Many gerontechnology research studies deal with the design processes and methodologies [8]. Both participatory design and codesign approaches speak to the need to include participants in the exploration of a design challenge and the subsequent design process. One of the challenges older individuals face is that technology has not always been designed with their specific wants or needs in mind [10]. A participatory design approach can bridge this challenge by including older persons in the design process of health and well-being technologies and services [11-13]. Studies on the implication of Internet of Things for future health care services and devices for older individuals position a participatory approach as critical [14,15]. As technology develops, there remains a need to explore the complexities of technology use in health care services intersecting with the older user [3,16], as well as the role of the older user in the design process [6]. It is also necessary to unpack the inherent tension among design research that allows for iterations, ambiguity, rapid prototyping, and health research that is hypothesis-driven and where evidence-based research is the norm [3]. This paper attempts to contribute toward the understanding of the design of technology-enabled services for the older user.

The aim of this study was to propose a theory that can be used when designing health and well-being services with the aging population as a target group. Findings from the study contribute a substantive theory to the service design body of knowledge, which explains the engagement of older individuals with Web-based services (including services that support health and well-being). The study adds value to Web-based health care service design practice by developing a deeper understanding of user perceptions and experiences within a sociotechnical context.

### Designing for an Aging Individual

Decreased physical mobility, eyesight, and cognitive processing may impact the QoL of older individuals. These individuals require support from family, friends, or the community to complete basic daily tasks and activities relating to their health and well-being. Technology and devices that support health care could offer alternative solutions to these challenges. These technologies can range from services that enable increased socialization (social media and communication applications) to health monitoring devices and emergency notification services.

The Web-based market for an aging population has been an area of research interest for a long period, but it has not yielded many insights into user-driven design in collaboration with older users [17]. This may be influenced by how aging users are viewed by both the service and goods providers, as well as the developers of health care and well-being technologies and services. A number of recent software solutions, apps and devices reflect the spectrum of interaction with aging individuals during the conceptualization and development of interventions and supportive solutions. These range from including aging individuals as end-user testers instead of cocreators [18] to collaborating with those close to the aging person (family and caregivers) [19]; finally, these also span to projects that include aging individuals in the process as cocreators [20].

The influence of age on the likelihood of engaging with technology is less extreme than once imagined [6,21], but the nature of, and influences on, the engagement of an older individual has been noted. Before the potential impact of health care technologies and services on the lives of aging individuals can be understood, the usage and perceptions of the aging population must be explored to identify possible barriers to participation.

From an economical perspective, it is crucial to consider the global growing aging community within our technological society [1,22]. If not considered during the conceptualization and design of products and services, it can be hypothesized that aging users who do not feel confident on use of the Web would cease to use the services that could possibly improve the support for their everyday activities and offer them greater independence [8,23].

### Technology to Support Aging in Place

Gerontechnologies offer technologies and innovations specifically designed for an aging community [6,24,25]. Another definition offered by Iffländer [26] is *age-based innovations*, which are defined as products and services specifically designed to acknowledge the needs of older users. Irrespective of the assigned title, these products and services focus on enabling an aging community to remain autonomous and contribute to a greater sense of well-being (including increased social engagement). A possible contribution of supportive technology and access to Web-based services is facilitating aging in place (AiP) of aging individuals.

A lack of understanding specific user needs can have a major impact on the innovation of various technologies that can support AiP [27]. This understanding must include a review of human factors that consider an individual’s limitations, his or her capabilities, as well as personal and cultural contexts [28]:

Only when the real needs of the elderly are correctly understood by innovators, fully specified in AiP digitalization, together with stakeholders’ inclusion in the innovation process and proper consideration of human factors and other contextual factors…can then ensure the success of AiP implementation [26].

To encourage continued engagement among aging users who can benefit from technology that facilitates AiP, the nature of their Web-based interaction, as well as the process of learning how to engage, must be considered. Aging individuals and those who care for them will embrace technological products and services that support AiP [20].

## Methods

### Grounded Theory Method

Grounded theory (GT) emerged from the research and practice of Glaser and Strauss in 1967 [29]. Since then, 3 main streams of GT have developed. The first represents the original ideals of Glaser (often referred to as Glaserian GT), the second variation of the method was conceptualized by Strauss and Corbin (in response to the earlier Glaserian variation), and finally, Charmaz’s constructivist GT [30]. At the heart of each GT stream is the exploration of real-world situations through rigid analysis and documentation to gain insights, and it is not based on preconceived ideas or assumptions [31]. The types of data collection tools vary, but qualitative methods, such as in-depth interviews, are prominent. The information gained is analyzed through coding processes, followed by making sense of the complex data and finally coming to a cohesive theory grounded in the data.

The process aims to conceptually explain how participants respond to a certain concept, phenomenon, or challenge [32]. The development of the theory is based on 3 foundations: constant data analysis (where data collection can happen simultaneously), theoretical saturation (data are collected and analyzed until nothing new is discovered), and theoretical sampling, which facilitates the emergence of theory [33].

### Recruitment and Participants

Participants were recruited through a gatekeeper organization, which has a broad reach throughout the Western Cape and South Africa and attracts individuals from varying ethnic and socioeconomic backgrounds. Presentations were made to members to introduce the project and highlight the parameters of participation. The parameters were that the participants had to be over 65 years and have access to Web-based services through a personal or shared device. The study did not explore problems with connectivity or internet penetration within South Africa. Following the presentations, 23 individuals requested to collaborate on the study, out of which 13 were interviewed (Table 1). The 2 workshops were hosted with 15 participants, out of which 5 participated in the interviews before the workshop. Not all of the interviewed participants joined the workshops. This study thus featured a convenience sampling with regard to the open call for participation; however, all participants met the defined project parameters. For this reason, the sampling method aligns itself with the traditional theoretical sampling one would expect in a GT study.

Ethical considerations were a primary focus in the study, and recruitment was dependent on the participants’ ability to give informed consent. Furthermore, 3 aspects contributed to the concept of informed consent practiced in the project: (1) participation was voluntary, (2) the nature of the project (including all benefits and risks) was explained before commencement of the research activities, and (3) participants’ consent was *valid*. The validity of consent was defined through the work of Ratzan [34], which proposes that, “…the elderly research subject's actual understanding of the experiment be accurate and complete…” Ethical considerations in the project aimed to establish an empathetic grounding for interaction and placing the participant at the center of any consideration or project decision.

The research process was initiated with an open discussion with participants, during which the goals of the project were introduced along with research activities. Participants had the opportunity to discuss their concerns or excitement, from which comments were noted down on the *participant coding and information form*. The form was part of a research process map and toolkit document, which supported the gathering of informed consent and first stage research observations (Multimedia Appendix 1). To quote interviewed participants directly, in an anonymous manner, they were asked to select their own pseudonym. This allowed participants to select the name they wanted to be known by and shifted participant identification protocols associated with anonymity and research ethics into a more human-centered realm.

**Table 1:** Participant group and research activity participation.

Participant code (PC)	Pseudonym selected by participant (only for interviewed participants)	Age (years)	Gender	Device type	Participation in research
PC1	Victoria	76	Female	Mobile phone, tablet, desktop	Interview and workshop
PC2	Marie	72	Female	Mobile phone, desktop	Interview
PC3	Convict1	85	Male	Mobile phone, tablet, laptop	Interview
PC4	Cordelia	82	Female	Mobile phone, desktop	Interview
PC5	Susan	74	Female	Mobile phone, desktop	Interview and workshop
PC6	Ina	82	Female	Mobile phone, laptop	Interview
PC7	Doll	65	Female	Mobile phone, tablet, desktop	Interview
PC8	Hellet	78	Female	Mobile phone	Interview and workshop
PC9	Cody	76	Female	Mobile phone, desktop	Interview
PC10	Diana	69	Female	Mobile phone, desktop	Interview
PC11	Leonie	71	Female	Mobile phone, tablet, desktop, laptop	Interview
PC12	Dick	69	Male	Mobile phone, desktop	Interview and workshop
PC13	Taffy	74	Female	Mobile phone, desktop	Interview and workshop
PC14	N/A^a^	68	Female	Mobile phone, tablet	Workshop
PC15	N/A	66	Female	Mobile phone, desktop	Workshop
PC16	N/A	83	Female	Mobile phone	Workshop
PC17	N/A	65	Female	Mobile phone, laptop	Workshop
PC18	N/A	67	Female	Mobile phone	Workshop
PC19	N/A	71	Female	Mobile phone, desktop	Workshop
PC20	N/A	77	Female	Mobile phone, laptop	Workshop
PC21	N/A	70	Female	Mobile phone, desktop	Workshop
PC22	N/A	77	Male	Mobile phone, tablet, desktop	Workshop
PC23	N/A	79	Female	Mobile phone, desktop	Workshop

^a^N/A: not applicable.

### Data Collection and Initial Analysis and Coding

The project followed a systematic process with regard to the collection and analysis of data in-line with GT. Data collection and analysis happen simultaneously in GT. The process of data analysis progressed through 3 main analysis cycles: initial coding, focused coding, and finally, theoretical coding. The initial coding cycle is rooted in iterative data collection and analysis. Once no new codes emerge as new data are analyzed, the focused coding and theoretical coding processes are completed. Data analysis was completed through line-by-line review and coding. ATLAS.ti, a Computer Assisted Qualitative Data Analysis software, was used to collate and analyze the data gathered. A key aspect of the software that assisted in the analysis of the data was its ability to code both abstractly, as well as in vivo. Using in vivo codes helps preserve participants’ reactions and the meanings of their views and comments.

**Figure 1:**
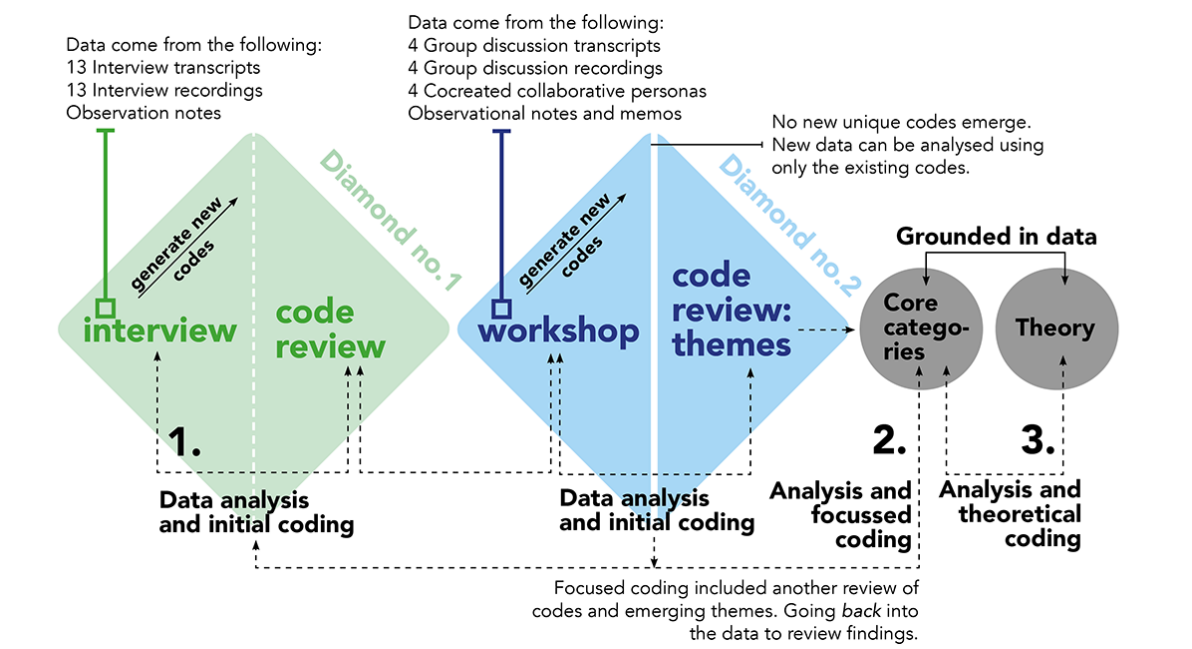
Data collection and analysis process.

The data collection and analysis process mirrored the *double diamond* [35] design process (Figure 1). In the first diamond, 2 phases were completed: (1) the gathering and analysis of interview data and observational notes, followed by (2) the reflection and code review phase. Interviews were a critical method in this study as they allowed the researcher the opportunity to listen and reflect on experiences of older individuals. Participants could decide on where it was most convenient for them to meet, resulting in a number of interviews being conducted in participants’ homes (10 in total); however, the remaining 3 interviews were conducted in a café. Participants were interviewed by a single researcher, who recorded the whole interview for transcription. To encourage a more open approach to interviews, participants were given contact details (email and phone number) to allow them to stay in contact with the researchers. If they wanted to share more information at a later stage, or ask a question, they could engage directly with the researcher.

At the interviews, participants were asked to respond to the following questions:

- How do you use Web-based services in your everyday life?
- How do you feel about technology and Web-based services?
- What, if any, are the challenges you have noted when engaging with Web-based services?
- Would you want to engage with technology and Web-based services? If yes, what would you like to see and how would you want to engage with devices and Web-based services?

The driver of each interview was to develop a narrative around the participants’ perceptions and experiences, and thus the questions were not considered as fixed. The interviewer remained open to conversational shifts as participants shared relevant stories. The process of *initial* coding during the analysis of 13 interview transcripts and observational notes was iterative, in that new data were compared with reviewed and previously coded data. Interviews ranged from 65 min to 93 min in length.

**Figure 2:**
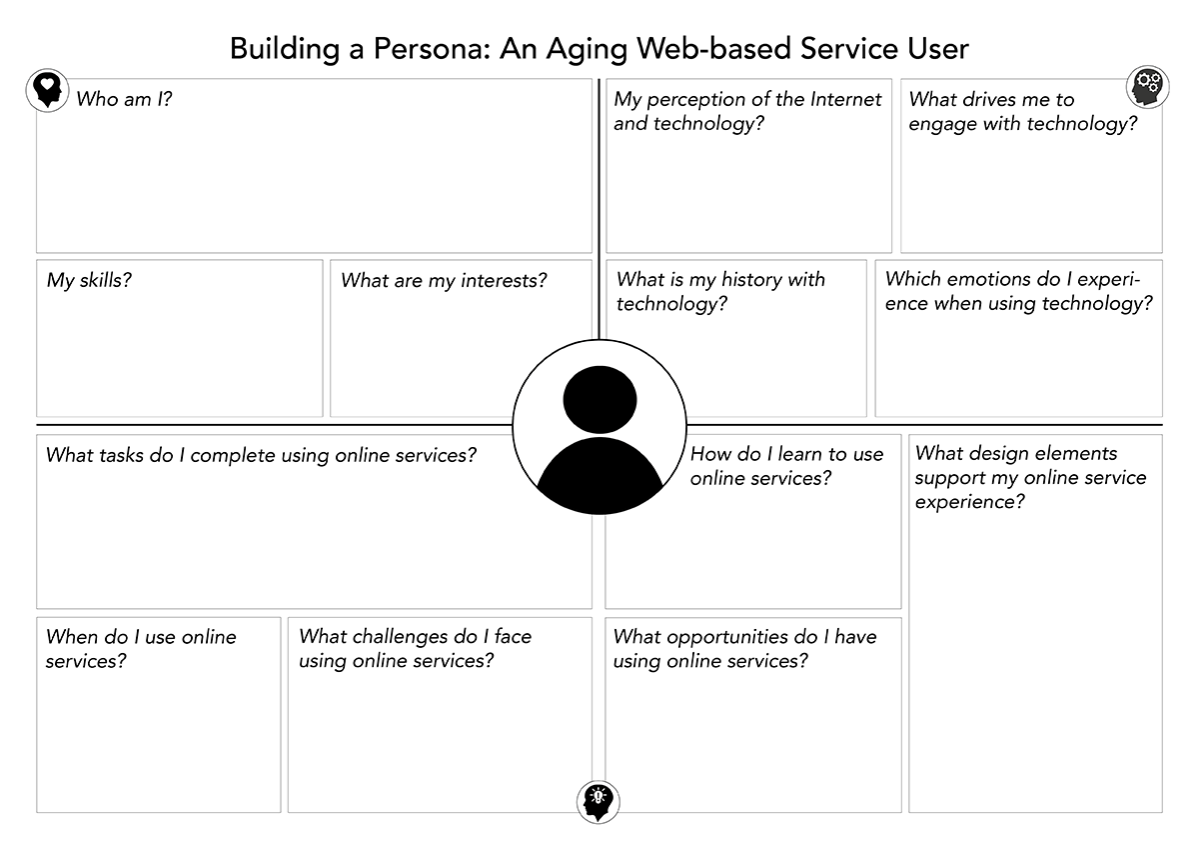
Collaborative persona template.

The emerging codes from the interview data analysis were seen as *provisional*, as they changed and evolved as more data were coded and compared. In the initial coding phase, the emphasis was on extracting data from interviews, comments, and observations captured during interactions with participants. The open process of initial coding yielded 155 individual codes. Following the code review, the number of codes decreased to 130 codes. This was the result of code-to-code analysis and the merging of conceptually similar codes. The second diamond also comprised 2 phases: (1) the gathering and analysis of workshop data (transcripts, observational notes, and workshop materials), followed by (2) the reflection and code review phase. In total, 2 workshops were completed, one with 7 and another with 8 participants. The workshops included 3 activities. The first activity was an introduction to a range of Web-based services, demonstrated on different devices and operating systems (Apple, Windows, and Android). At the interactive demonstration session, participants shared their own experiences. The second set of activities focused on the creation of a collaborative persona. The collaborative persona template (Figure 2) served as a discussion catalyst, probing the groups of participants to respond collaboratively.

**Figure 3:**
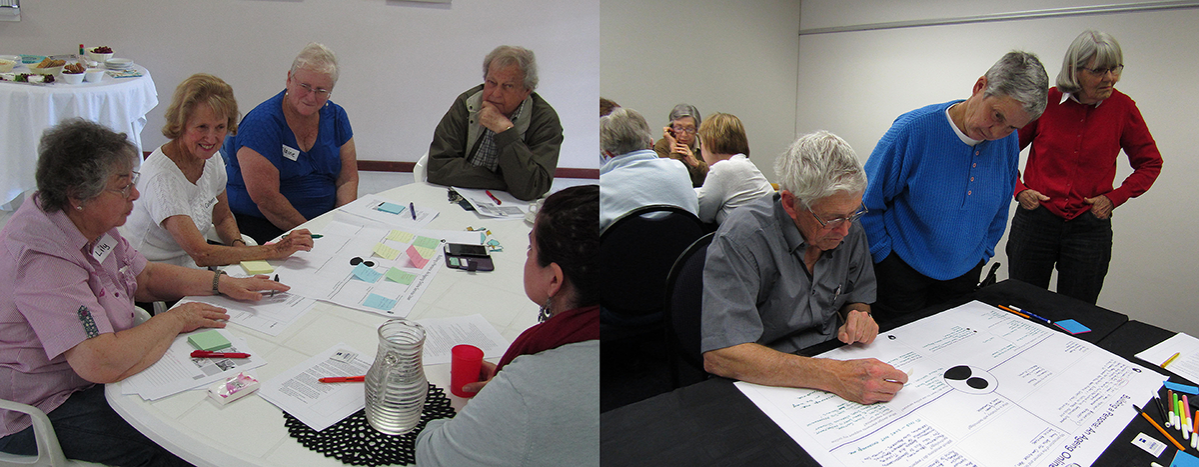
Participants and worksheets to support discussion and collaboration (workshop materials).

Several key factors were highlighted through the activity. The discussions were recorded for transcription and analysis. The template and group discussion recording allowed for the gathering of experiences and perceptions from 3 distinct perspectives: the person, the person and technology, as well as the person and Web-based services (Figure 3). Each workshop group was facilitated by a design researcher who observed interactions and posed probing questions during group discussions and cocreation of personas. The paper-based personas were digitized following the workshops (Multimedia Appendix 2) and shared with workshop participants. The final workshop activity focused purely on the participants’ needs. All facilitators and technical support worked with individual participants to answer questions regarding Web-based experiences, set up access where needed, or offered device support where possible.

Following the workshops, *initial* coding continued. Workshop materials (in the form of 4 cocreated personas), discussion transcripts for each of the 4 working groups, and observational notes were analyzed and coded. During the analysis and coding of data from the second workshop, no new codes emerged. The analysis and coding of workshop data were followed by a review of the captured initial codes. As part of the review, each code was assigned an introductory word or phrase, which identified the larger focus area and the theme of which the code was a part (Table 2).

**Table 2:** Code groups resulting from code review.

Introductory word or phrase	Code group description
Cognitive impact	Codes relating to the positive and negative impact of using technology and Web-based services on cognitive functions.
Design	Codes relating to the impact of design and interface on the user experience of technologies and services.
Emotive response	Codes relating to both positive and negative emotions experienced by participants when engaging with technologies and services.
Infrastructure and affordability	Codes relating to the affordability of Web-based access and the infrastructure that supports it in South Africa.
Learning facilitation	Codes relating to the method, nature, and process of learning as experienced by the users.
Nature of the internet	Codes relating to the scope and nature of the internet.
Perception	Codes relating to how users perceive technologies and services: the benefit, value, challenges, and expectations.
Physical and digital	Codes relating to the intersection of the physical and digital space and when participants identified comparisons between the two.
Privacy and security	Codes relating to concepts of safety on the Web, Web-based threats, and privacy.
Reason for adoption	Codes relating to reasons for adoption of technologies and services.
Using services	Codes relating to actual technologies and services being used and the experience of specific service elements.
Interaction	Codes relating to all aspects of user interaction with technologies and services from both a positive and negative perspective.

Subsequent to the code review, a final list of 135 codes was defined. The code review process informed the next data analysis phase within the grounded study. The focused coding process critically reviewed each code and the emerging code groups, in relation to the theme identified throughout the data gathering process. The focused coding process resulted in a set of core categories.

### Focused and Theoretical Analysis and Coding

Through a systematic review and analysis of codes (in relation to original data, initial codes, and identified themes), the focused coding process resulted in a set of core categories. All initial 135 codes were absorbed within the emerging core categories, and they reflected a more conceptual connection and relationship of themes noted during the code review. The core categories descriptions are grounded in the original comments and perceptions shared by participants. Through the coding process, the data gathered were deconstructed into essential elements, reviewed for patterns and relationships, and then constructed into more complex concepts. The core categories are *digital context*, *cognition and learning*, *emotive response to Web-based interaction*, *user context*, *perceived benefits*, *nature of user interaction,* and *design to support use*.

#### Digital Context

Aging users viewed technology as products and services that allow access to both convenience and information. Participants felt that products, apps, and services were not designed specifically with aging users in mind. Factors noted in this study that impact user engagement and willingness to interact relate to personal perception, emotional responses, and how each individual views his or her own ability to learn and master new technology.

#### Cognition and Learning

Diverse learning networks were noted in the study. When new skills, ways of working, and ways of learning are introduced, users must adapt their cognitive understanding to engage with the process. Cognitive ability plays a role not only in an aging user’s willingness to engage but also in the long-term usage. Olphert and Damodaran [23] hypothesize that the difficulties that aging users face on the Web may be because of the complexity of technology combined with the cognitive load required to engage actively. In this study, participants mentioned 3 other main learning scenarios: learning from peers, learning from family members, and exchanges with external individuals (such as technical assistants at shops). The engagement with others resulted in mutual learning, during which the aging individuals expanded their knowledge and skill base, whereas those they interacted with developed a keener understanding of the challenges and opportunities that aging presents.

#### Emotive Response to Web-Based Interaction

Both positive and negative emotions were observed when participants discussed engaging with new technologies. One of the key emotional triggers noted by participants was that they often felt *forced* to engage with new technologies and services. The sense of frustration that they felt was heightened if their immediate network either could not or chose not to offer support and tutelage. Hellet (a pseudonym selected by the participant) noted the following:

I really think I need to go for lessons. My daughter always says “what lessons? Just sit and try” like I’m not trying in the first place.

These early reactions to the use of a Web-based service can influence the way that older users experience the technology as a whole.

#### User Context

The requirements of aging users are often overlooked by developers and vendors as they do not play an active role in the conceptualization or design of Web-based services and other technologies [36]. Participants in this study acknowledged that they should play a greater role in becoming computer and device literate, to enable them to engage with new technologies and services with more confidence. Cordelia (a pseudonym selected by the participant) noted the following:

Programs are often designed by people who are too clever. Sometimes, I don’t know what’s going on, then what do you do? Explain to people. As we work, we learn...I need to learn by doing it myself.

Acknowledging the unique personal context of an aging user is a key factor in facilitating Web-based engagement. The impetus to engage, the perceived value of Web-based engagement, and the design characteristics of an enabling environment must all be considered.

#### Perceived Benefits

The rise of Web-based activity among aging users is linked to their perception of services, technologies, and apps as tools to support everyday activities. One participant (with the pseudonym Victoria) noted the following:

So the value is when you have limited mobility, when you're housebound…You could have your Skype, which you can use to check in with your neighbors. If you are housebound and you can contact your friends or family. You can phone your neighbor. So the access is absolutely brilliant. You have unlimited access, you can access anybody, your children, anybody and let them know your situation.

In the context of an everyday tool, supportive devices and services can offer users clear value propositions. Digital technologies and Web-based services play a major role in addressing the so-called *burden of care* often associated with an aging population [23]. Participants stated that Web-based access could benefit an individual by allowing access to emergency services, lessen feelings of loneliness and isolation, and benefit individuals with limited mobility. Access and support provided through technology could extend aging individuals’ ability to age in place, allowing them to age in their own home even though various physical, cognitive, or social capacities may decline with age [26].

#### Nature of User Interaction

Once the perceived value of the interaction is high enough for an aging user to engage, even though he or she may have negative perceptions, the nature of the interaction space and platform may still impact continued engagement. In many cases the emotions noted included frustration at one’s own inability to understand and successfully navigate a Web-based service and supportive devices, the fear of the unknown, and feelings of frustration when forced by family or community members to engage with digital technologies. These emotional reactions form part of the human experience of a technological interaction. Digital interaction and the design of this interaction may influence the user experience, as well as the process through which the user learns to navigate the interaction. If not considered during the conceptualization and design of Web-based services, it can be hypothesized that aging users who do not feel confident would cease to use the services that could possibly improve support for their everyday activities [23]. This result has led researchers to define a *fourth digital divide*, which is not characterized by a lack of access or skill but rather by a lack of clear motivation or interest [23].

#### Design to Support Use

Using devices and services does pose a number of challenges to aging users, including limited experience with devices, limited experience with Web-based services, or a lack of interest [37]. It is not only the design of the system that needs to support interaction and learning but also the design of interfaces and interactions. The applied practice of designing for aging users and creating more accessible design have received much attention from various projects and researchers over the past decades [37-42]. In addition to individual researchers, various projects have investigated accessibility from an aging perspective, including the *Web Accessibility Initiative: Ageing Education and Harmonisation* [43]—a European Commission Specific Support Action project (Information Society Technology 035015). Overwhelmingly, participants stressed the need for design elements that favor simplicity; however, this may not be an easily generalizable standard. One of the primary reasons for ending one’s use of Web-based services has been described as *excessive complexity of the technology* [23]. Participants said that they preferred clear action buttons and a simple style of communication.

During the focused coding process, memos allowed the researcher to keep track of considerations and decisions. The writing up of memos also provides for a structured moment to pause and reflect on the process, encouraging a holistic reflective practice. Finally, the process of theoretical coding responded to the 2 coding aims stated by Flick [44]. First, the process aims to clearly understand and explore the research context or question. Second, it identifies the relationships among categories or components extracted from what was found.

During the theoretical coding process, the emergent core categories were reviewed from a structural perspective to understand the relationships between categories and the nuances at play when considering core categories as part of a whole and not individual sectors. The result of the theoretical coding process was the substantive theory, which explored the process and factors that impacted an aging individual’s willingness to engage with technology and services. Saturation was explored at data level (when no new codes emerged through constant comparative data analysis) and again at theoretical level (when the emerging theoretical constructs, literature, and memos were compared and yielded no new variations). Through the process of theoretical coding, these categories formed the foundation for the emerging theory of *Ageing User Decision-Driven Engagement* (AUDDE).

## Results

**Figure 4:**
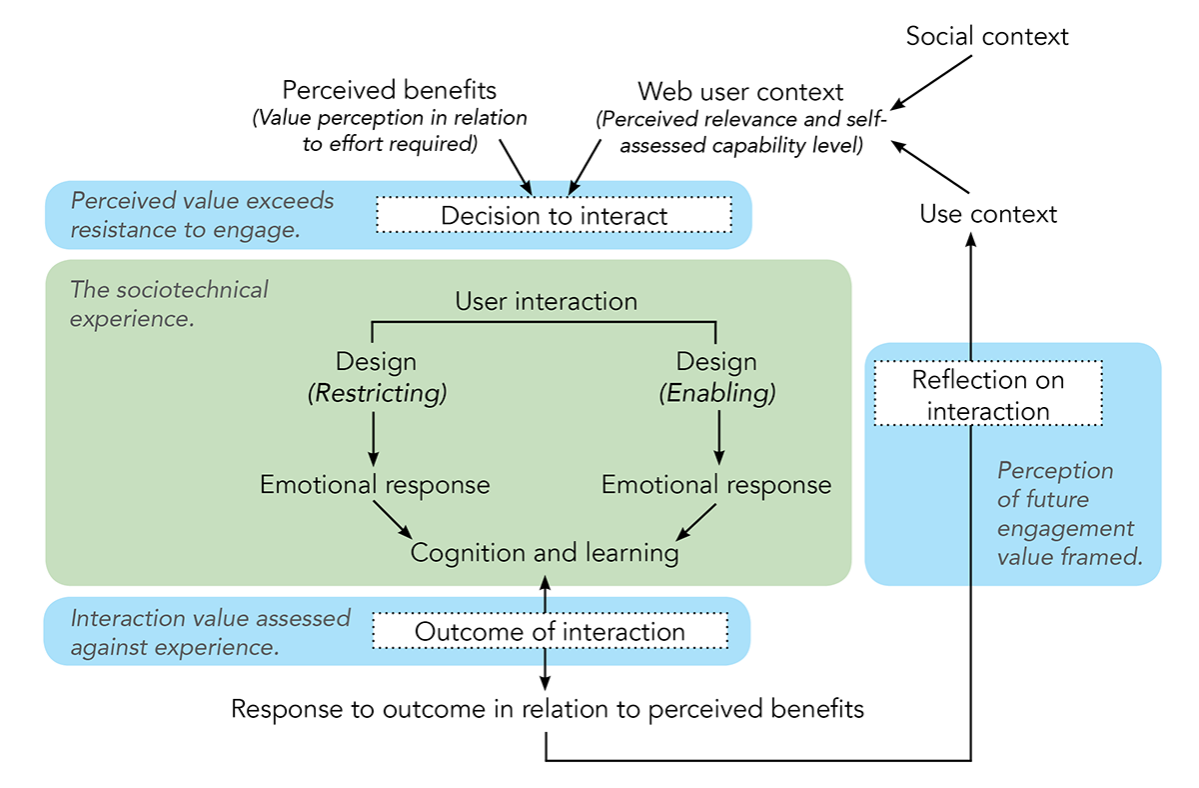
The theory of AUDDE: Ageing User Decision-Driven Engagement.

The result of the study was a grounded, substantive theory that explores the factors, and process, of aging individuals engaging with services on the Web. The term *cyber-seniors* refers to 2 emerging groups of aging users, the first is that of the *technology lovers* (who engage willingly and are fascinated by technology) and the second is that of the *technology users* [45]. Technology users see technology as a tool to achieve a specific goal. Participants who took part in this study were overwhelmingly *technology users*. Only 1 participant in the study could be defined as a technology lover; all other participants commented on the *tool* nature of Web-based services or their ability to *help you do something*. AUDDE (Figure 4) highlights the decision-determined engagement that characterized the aging technology users within this study.

The theory proposes an iterative process in the decision-making cycle. When aging users decide to engage with a supportive device, app or service, or decide not to engage, 2 main factors form the basis for their ultimate decision. These factors are defined as *perceived benefits* and *Web-based user context*. For a user to engage, the perceived benefits must outweigh any hesitation that forms part of the Web-based user context. The perceived benefits must be made clear through a value statement. This value statement could be found in the form of an advertisement, but among the aging, it will more likely be word of mouth or the suggestion of a trusted medical professional or a similar individual. Family members and peers are the most likely candidates to share a value statement with an aging individual. Once the individual is aware of the perceived benefit and value that an engagement may have, the decision to interact is dependent on the level of resistance within the user context. The Web-based user context is shaped by 2 spheres of influence, the *social context* and the *use context.*

The first sphere of influence that impacts the decision to engage is the user’s *social context*. Here, the social context refers to the perceptions of the aging individual’s social group, communities of interest, and communities of practice with regard to the technology. Within these social constructs, the aging individual may feel pressured to share the communal peer point of view of his or her social groups. The view of individual family members or friends has a similar ability to shape the perspective of an aging individual. The emotional and social influences on the willingness of an individual to interact with a supportive technology or service are crucial to potential engagement. Equally crucial is the emerging and constantly evolving *context of use*.

The use context of aging individuals is informed by every interaction they have had with the device or service in question, and it may even include a broader range of technological interactions. Every interaction contributes to an individual’s perception of ease of use, cognitive demand, convenience, and overall advantage. In this way, the use context is constantly evolving. The context of use is formed through a process that occurs when the user has decided to interact and revolves around 2 process points, the *outcome of the interaction* and *reflection on interaction*.

The outcome of the interaction is a process of evaluation that the user completes at the end of a task. The task does not have to be completed successfully, or even completed at all, for the outcome of the interaction to be evaluated. The design of technologies, devices, and services plays a pivotal role in user interaction. Services that take into account different levels of physical ability and focus on enabling simple and specific tasks are of greater value to aging users. Design choices that create an enabling experience elicit a positive emotional response from users. These emotions include pride in one’s ability to complete the task unaided, a heightened sense of accomplishment, and joy. Given the nature of the activity, the completion of the task may also elicit a sense of relief. A design that restricts task completion elicits feelings of frustration and confusion. The emotional reaction to a task can often permeate the entire interaction. If aging users, for example, struggle to switch on the device or use it as intended, their initial feeling of minor annoyance can be aggravated by subsequent challenges. The emotional reactions experienced throughout the interaction affirm or challenge perceptions which the user had before. A positive experience may challenge a personal resistance to engage or strengthen previous beliefs that engagement has value. A negative experience may call into question previous perceptions relating to the value of engaging or reinforce previous resistance to engagement.

Linked to the emotional experience of aging users, while interacting with a Web-based service, is the cognitive experience and the potential for learning. Both physical and cognitive decline are key markers of the aging process; however, both manifest in varying degrees and escalate differently among the aging community. Products and services, which offer guidance in the case of possible lapses in user memory or provide a step-by-step task guide, support the unpredictable nature of cognitive ability among this user group.

The learning process for aging users is mediated by either an external stakeholder (a friend, a family member, or a professional encountered when seeking help) or self-exploration. These learning experiences may happen before engagement or may take the form of coached support while using a device or service. How an aging user interacts, the cognitive experience of the interaction, and the emotional consequences and resulting learning process are all interwoven elements of an iterative cycle. A user may go through multiple cycles within this sociotechnical experience before reaching the end of the interaction. The outcome of the interaction could be a successful task completion, an unsuccessful task completion, or an interrupted task (ended before either a successful or unsuccessful task completion). Irrespective of the nature of the outcome, the experience of the interaction will result in the user’s reflection on the perceived value expectation in relation to the interaction experience. This process will inform the future *use context*.

## Discussion

### Involving Users in the Design Process

When reflecting on the emerging AUDDE theory, the complex nature of the networked society becomes clear. Knowledge and experience within the Web-based and digital service realm transcend single disciplines to create service systems:

Actual service systems can be described as complex sociotechnical systems, being approached in an interdisciplinary vision that integrates business functions, technology, and human resources, with the final aim of creating value and benefit through the generated services.

46

Acknowledging this complexity is crucial when conceptualizing and designing devices, technologies, app, and Web-based services, which aim to support the health and wellness of aging individuals. Researchers and designers must systematically review both the technical and social systems that are at play during an interaction [47]. The technical systems contribute engineered interaction spaces that are designed to be anticipatable and reliable. The social systems are in many ways dependent on the technical systems and evolve throughout interaction encounters. The fast pace of technological development today requires that we endeavor to gain a greater understanding of social systems, to navigate new sociotechnical interactions as they emerge [48].

End users are social beings, who evolve and grow. As such, it is impossible to define social value as a constant, and the social impact on interactions must be considered within the *more institutionalized traditions or regulations inside various user communities* [49]. To understand sociotechnical interactions, it is important to understand that the technology and social context develop reciprocally [50], as well as know the value system that these relationships represent. In AUDDE, the perceived value of the interaction is a crucial catalyst for engagement. Aging users continuously make meanings of their experiences, which affect their current and future actions.

### Limitations

It must be emphasized that AUDDE is firmly grounded in the perceptions and shared stories of a specific group of aging users living in Cape Town, South Africa. The relevance of this theory to aging users’ experiences outside this geographic area must be verified. The regularity of participants’ use of supportive technologies was not a consideration within the delimitations of the study. AUDDE is a substantive theoretical contribution to the body of knowledge of design, but it has the potential to be explored within the context of other studies. To ensure that the emerging theory represented the views and shared experiences of participants, an interactive discussion session was conducted with a participant who took part in the interview and workshop. The session was discussion driven and presented the research process and emerging theory before requesting feedback and comments from the participant. The feedback supported the emerging theory, but feedback from a larger audience will be beneficial.

### Conclusions

As the global health care systems become strained with growing aging populations [1], technology may facilitate alternative ways of engaging with and supporting older individuals. Health and well-being products and services draw on the potential of Web-based interaction and technology to support independence and AiP. Product and service design projects and initiatives encourage contributions from aging users to varying degrees. Some projects position older individuals as final validators of a product or service. In this case, input and feedback from the older individuals are gathered subsequent to the development of the artifact or service. Projects that focus on a human-centered approach position older individuals as central to the design and conceptualization process [3]. This approach allows for continuous feedback and interaction between the design team and the envisaged end users. AUDDE contributes to the theoretical body of knowledge in design and aims to explain how and why aging users engage with technology. The theory proposes an iterative cycle of evaluation during which users decide to engage when the perceived value of the interaction is greater than the perceived challenge of interacting. If health care products and services are to be contextually relevant and thus viable, aging individuals must be included in the design process.

## Appendix

Multimedia Appendix 1 Research process map and toolkit.

Multimedia Appendix 2 Digitized collaborative personas.

## Abbreviations

AiP: aging in place
AUDDE: Ageing User Decision-Driven Engagement
GT: grounded theory
QoL: quality of life
