# Supplementary material for: Engaging Aging Individuals in the Design of Technologies and Services to Support Health and Well-Being: Constructivist Grounded Theory Study
Source: JMIR Aging. 2019 Mar 20;2(1):e12393. doi: 10.2196/12393 (PMC6716485; doi:10.2196/12393)
Supplement: Multimedia Appendix 1 [file aging_v2i1e12393_app1.pdf]

Data & Coding Map

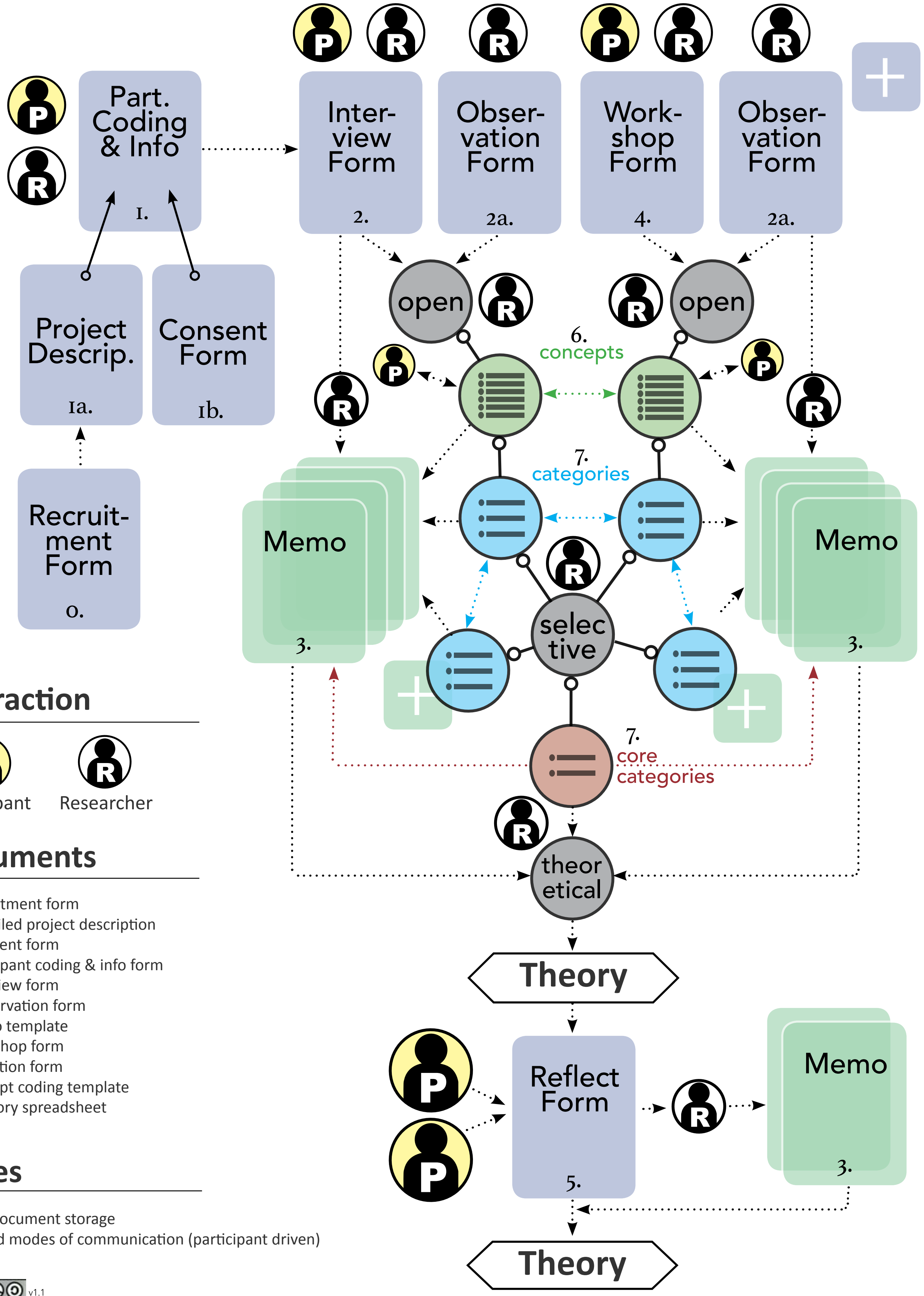

Interaction

P Participant R Researcher

Documents

0. Recruitment form
1a. Detailed project description
1b. Consent form
1. Participant coding & info form
2. Interview form
2a. Observation form
3. Memo template
4. Workshop form
5. Reflection form
6. Concept coding template
7. Category spreadsheet

Notes

Secure document storage
Preferred modes of communication (participant driven)

cc BY NC SA v1.1
© Vikki du Preez

Form 1: To be completed with the participant.

Participant Coding & Information

Participant Code:

Pseudonym:

Research Activity:

Date:

Full Name: ______________________________

Gender: ______________ Cell Number: ______________

E-mail: ______________________________

Age ______________________________

Step 1: Talk about the project ☐ *tick if completed*

Introduce the project, yourself and the goals of the study. Talk about why you are doing this and what excites you about the project. Explain what the participant can expect and what research activities you hope they will participate in. Give the participant a description of the study to keep.

Step 2: Talk about the person ☐ *tick if completed*

Ask the participant if they have any questions or concerns. Note down any that they may have and answer their questions.

Step 3: Talk about the process ☐ *tick if completed*

Introduce the research activity. Explain any tools that you will be using. Let the participant know the approximate length of the activity. Restate that all activities are voluntary and that they are able to stop participating at any point during the session.

Step 4: Talk about the protocol ☐ *tick if completed*

Introduce the ethics process and consent form. Walk participant through the form. Note down any concerns and how you addressed them.

cc BY NC SA v1.1
© Vikki du Preez

Form 2: To be completed by researcher.

Interview Form

Pseudonym:	Participant Code:	Date:
XXX	XXX	X/X/X

Copy transcription text in block, identify concepts on the right. Use as many pages as required.

No. of Pgs. X/X

Transcript Text	Open Code

CC BY NC SA v1.1
© Vikki du Preez

Form 2a: To be completed by the researcher.

Observation Form

To be used in conjunction with other recording methods. Note down key aspects that you observe before or during the research activity, or upon reflection. Indicate if you have asked the participant(s) about something you have noted.

Participant Code:

Research Activity:

Date:

The Person

Level of engagement, emotions, reactions to questions, body language etc.

The Place

Context, environment etc.

The Research Tool

Faciliatating interaction, understandable, etc.

The Researcher

Behaviour, learning, reflective practice, etc.

v1.1
© Vikki du Preez

Form 3: To be completed by the researcher.

Memo

Ensure that you link memos to participants, forms or resources.
Reflect on your memo to extract the key notes and capture them.

Memo Date:

Research Activity:

Participant(s) referred to in this memo:

What is/was the catalyst for this memo?

Event, idea, observation, data comparison, theory implication etc.

Memo:

Key note(s) from memo:

CC BY NC SA v1.1
© Vikki du Preez
