# Supplementary material for: Engaging Aging Individuals in the Design of Technologies and Services to Support Health and Well-Being: Constructivist Grounded Theory Study
Source: JMIR Aging. 2019 Mar 20;2(1):e12393. doi: 10.2196/12393 (PMC6716485; doi:10.2196/12393)
Supplement: Multimedia Appendix 2 [file aging_v2i1e12393_app2.pdf]

User Created Persona:
An Ageing Online Service User

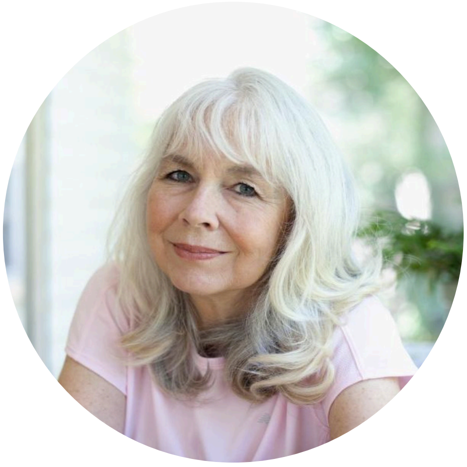

Stephanie, 70

Who am I? My skills? What are my interests? History with technology?

I'm a widow who has been described as gregarious, a good communicator, community minded and an extrovert. I live by myself with my beautiful dog! I have two children, the first is my daughter who lives in Canada with my granddaughter. My son lives in New Zealand with my two grandsons. After my husband's death I had no family left in South Africa but my friends are my support group. I love to keep busy and volunteer at a number of public services, including knitting for the Clothing Guild. Other interests include gardening, birding and being outdoors. I absolutely hate shopping!

My perception of the Internet and technology?

I have a positive attitude towards technology and use it on a number of devices including my phone and desk top computer.

What drives me to engage with technology?

A key thing is being able to communicate with my kids and grandchildren. I felt forced into online banking as I need to do EFTs.

Which emotions do I experience when using technology?

I'm a bit of a gadget person and love a challenges. There is great joy when I can chat to my family or see pictures of my grandchildren. But, technology is still cold and sometimes I'm not encouraged. I often feel irritated with the advertisements or frustrated when I can't figure something out.

PRACTICAL USAGE

What tasks do I complete using online services?

Online banking, booking of travel plans (easier than travel agent), finding information (ask.com), checks weather, local news, sharing photos (family and grandchildren), emails & Skype (communication) as well as games and buying airtime/ electricity.

When do I use online services?

Everyday I access news, weather and email. Monthly Skype with family.

How do I learn to use online services?

My daughter can answer questions as well as friends. Friends share new online services and ideas. Not comfortable asking staff at computer/ technology shops.

PERCEIVED OPPORTUNITIES & CHALLENGES

What challenges do I face using online services?

I am worried about security and how to be safe online. Changing (updating) interfaces is challenging and creates confusion. Threat of viruses is challenging.

What opportunities do I have using online services?

Mainly I believe the opportunities to be convenience (making every day tasks easier) as well as online shopping. Allows access to services should something happen to me (be indispensed).

What design elements support my online service experience?

A bigger font. Less visual clutter on websites (difficult to know what is important). Consistency with website appearance (less unneeded updates). Steps and processes need to be clear (MUST provide clear steps and feedback during the process).

User Created Persona:
An Ageing Online Service User

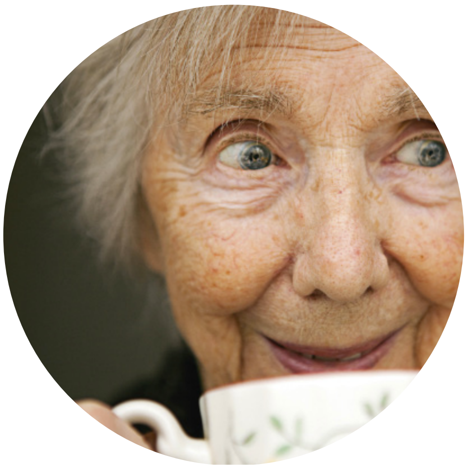

Astrid van Jaarsveld, 71

Who am I? My skills? What are my interests? History with technology?

Astrid is a divorced woman living in Cape Town. She has a daughter (40) who lives in the UK and a son (38) who lives in Cape Town. Astrid also has 3 grandchildren (two girls ages 5 and 3, and a baby boy). She is a social person and an extrovert who belongs to a book club and loves to travel. Astrid used to be an administrator and still works part time. She is reasonably patient. She is good with numbers, and loves handcraft, movies, brain games and cooking. Astrid lives by herself in a cottage. She used basic office software and email in her working life, but went online more when her children left home. Astrid is very interested in new technology.

My perception of the Internet and technology?

An essential tool. Scary in the beginning but easier if you use it often. There is just "too much information!" Prefers to access the Internet from a desk top - the size of a tablet is a problem.

What drives me to engage with technology?

Communication with family is a key driver. Banking. Booking tickets, the convenience. Information and news being accessible. Sharing made easy: photos and file transfers.

Which emotions do I experience when using technology?

Keen to explore and love the convenience, but I'm nervous, even fearful. Especially with financial transactions.

PRACTICAL USAGE

What tasks do I complete using online services?

Online banking, YouTube, online shopping (Takealot, Loot), social media (Facebook, family and friends), online communication (Face Time and Skype).

When do I use online services?

Daily for work and pleasure/communication.

How do I learn to use online services?

My son can help me, but my daughter isn't too tech savvy. Mostly I learn by trial and error. You need a very patient person to help you.

PERCEIVED OPPORTUNITIES & CHALLENGES

What challenges do I face using online services?

I am worried about security, and how to be safe online. Having to constantly change my passwords and user names for online services is challenging.

What opportunities do I have using online services?

I love the idea that so many different devices can talk to each other: P.C., iPad, smart phone, printer, scanner and copier, etc. All the different devices means that I have many opportunities.

What design elements support my online service experience?

Some of the key design elements that support my online activities: simplicity, unfussy design, size of text and font (san serif for clarity), colour (high level contrast), simple language, and use of audio support.

User Created Persona:
An Ageing Online Service User

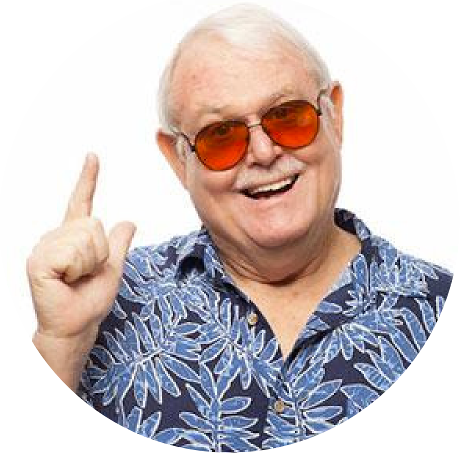

Horatio, 74

Who am I? My skills? What are my interests? History with technology?

I'm a 74 year young widower living in Cape Town. I'm not very active but in good health. I'm semi-retired and will be happy to find wife number three. I have three children, one in Cape Town, one in London and one is in Australia. All together I have 5 grandchildren, I don't have any pets. I still consult in the banking industry (my previous profession). I enjoy bowls, studying the financial markets, making models, collecting good whiskey and travelling. I have a working knowledge of technology but will always see it as a tool.

My perception of the Internet and technology?

I'm inquisitive and sometimes I'm curious but I'm happy with my current level of knowledge.

What drives me to engage with technology?

I enjoy being able to access information and maintain my work connections through email and online correspondence. I also enjoy being able to stay in contact with my children.

Which emotions do I experience when using technology?

I get extremely frustrated with slow connections.

PRACTICAL USAGE

What tasks do I complete using online services?

I'm okay with technology but it is not a focus for me. I use it as a tool. Mainly I check the markets, buy and sell stocks online, email, online tax and online banking.

When do I use online services?

Daily, first thing in the morning and last thing at night.

How do I learn to use online services?

When I'm stuck I ask someone around me; my children or grandchildren or a more tech savvy younger bank colleague.

PERCEIVED OPPORTUNITIES & CHALLENGES

What challenges do I face using online services?

I don't really see any challenge to not understanding technology. I live in a bit of a bubble. Sometimes the speed of my connection is annoying.

What opportunities do I have using online services?

I enjoy finding information about my hobbies, staying informed and planning travelling. I'm learning Skype and social media to connect with my family. I may even try online dating!

What design elements support my online service experience?

My biggest problem is too many small, unexplained symbols and codes as buttons. Explain things more clearly, or design things in a way that I can easily find more information or an explanation - maybe even in audio form.

User Created Persona:
An Ageing Online Service User

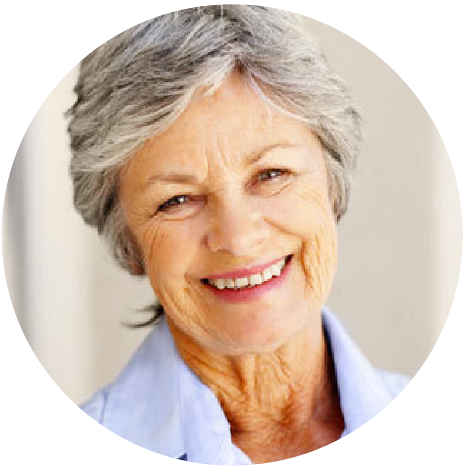

Jenny Johnson, 75

Who am I? My skills? What are my interests? History with technology?

My friends describe me as patient, organised, motivating, a good communicator and as a person's person. I enjoy watching sport, listening to music and reading. My musical taste includes playing the violin. I'm a widow with three children, a boy (50 years old) and two younger daughters (45 and 41). I am a teacher at heart and taught high school for many years. My main interests include cooking, gardening, photography and travel. I'm a comfortable leader and I'm self driven. My experience with technology is rather limited.

My perception of the Internet and technology?

I see the online space as challenging and informative. It is a bit strange sometimes as I did not grow up with technology and all it can offer.

What drives me to engage with technology?

Communication and convenience are the main drivers. A key thing though is that I'm afraid to be left behind. This is the way the world is going and I need to grow with it.

Which emotions do I experience when using technology?

Happy when technology allows me to see and connect with my family overseas. Exited at the prospect! Sometimes the experience makes me worried, anxious and impatient.

PRACTICAL USAGE

What tasks do I complete using online services?

Online banking, research via search engines (Google), email, online communication (Skype), travel services and booking, videos (how to do things) and shopping online.

When do I use online services?

Daily, generally in the late afternoon and evening.

How do I learn to use online services?

When in doubt I phone a friend. Mainly I learn through trial and error. I would love to attend more workshops. Repetition is key to learning.

PERCEIVED OPPORTUNITIES & CHALLENGES

What challenges do I face using online services?

I'm uncertain sometimes about what I should do - linked to a lack of knowledge. Sometimes the size of the keyboard and font visibility is a problem.

What opportunities do I have using online services?

Online access allows me to live and work more conveniently: shopping, banking, research, communication, administration and video communication (Skype).

What design elements support my online service experience?

Some of the key design elements that support my online activities: large text, clear highlighting, distinct colours, better guidelines as to why systems change (e.g. banking).
